# Supplementary figures and images for: The mechanism of MICU-dependent gating of the mitochondrial Ca2+uniporter
Source: eLife. 2021 Aug 31;10:e69312. doi: 10.7554/eLife.69312 (PMC8437439; doi:10.7554/eLife.69312)

MICU1

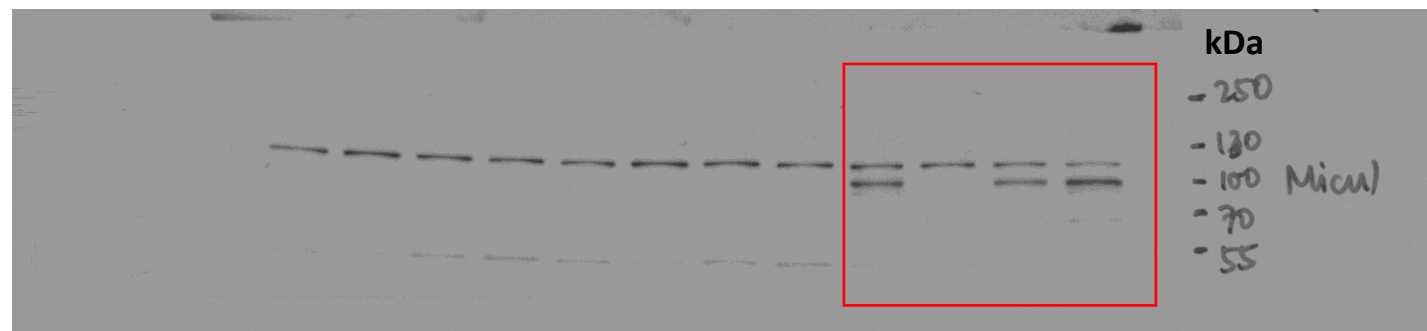

MCU

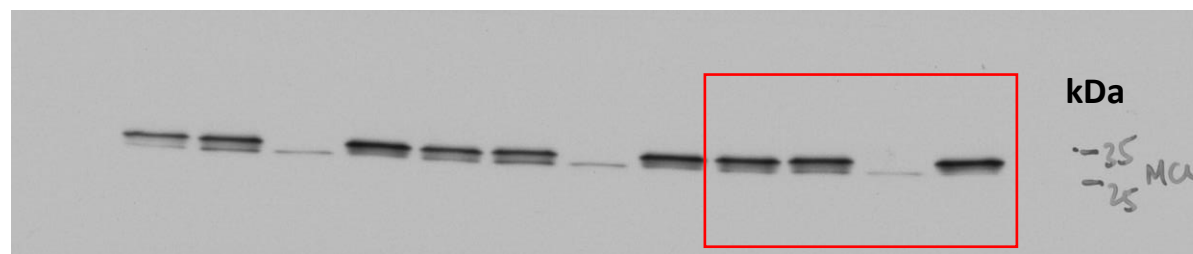

EMRE

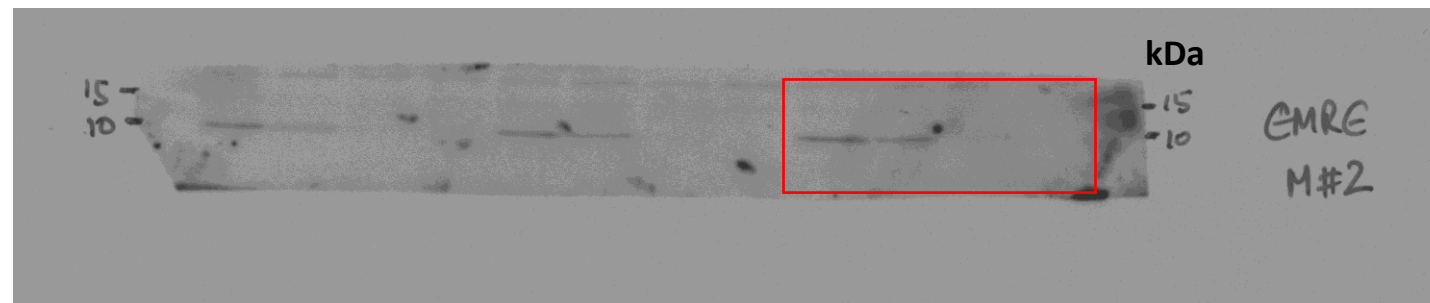

TOM20

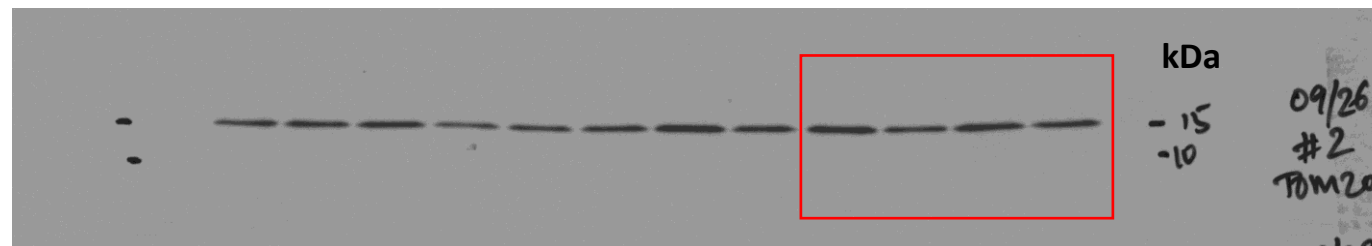

Figure 1—figure supplement 1—source data 1. Raw Western blot image for panel G.

Supplement: Figure 1—figure supplement 1—source data 1. [file elife-69312-fig1-figsupp1-data1.pdf]

**MICU2**

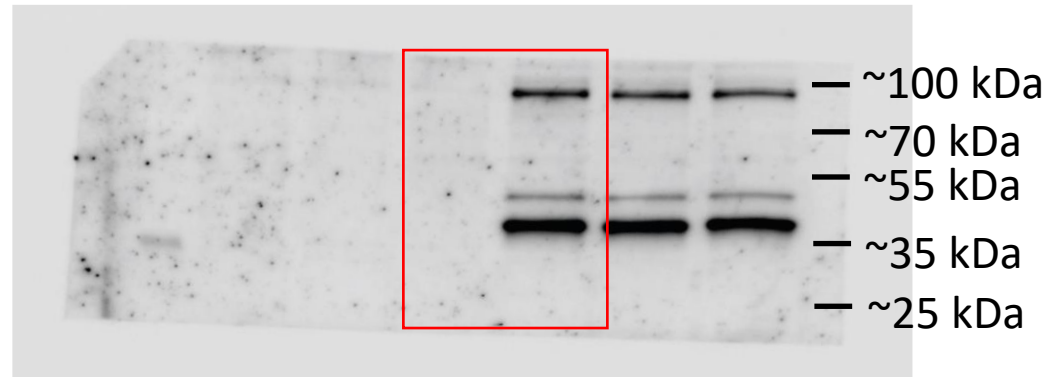

**TOM20**

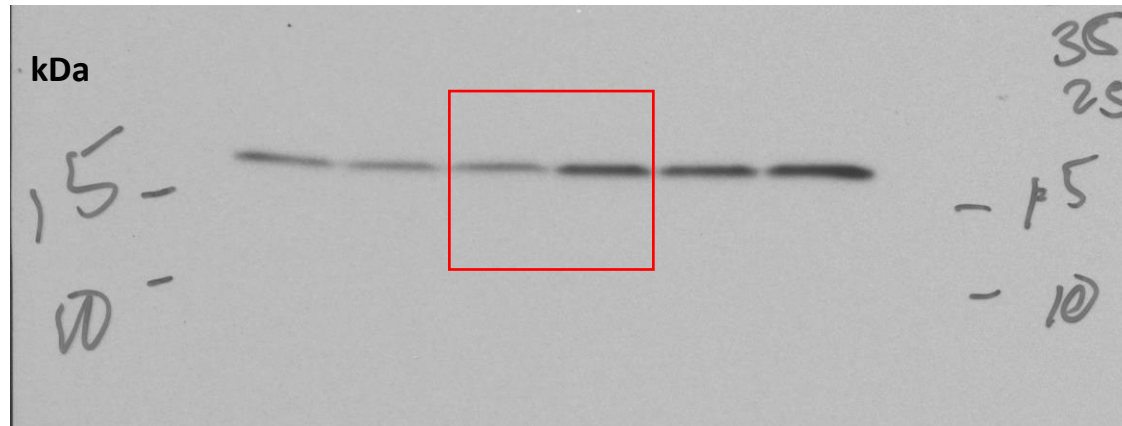

Figure 1—figure supplement 1—source data 2. Raw Western blot image for panel H.

Supplement: Figure 1—figure supplement 1—source data 2. [file elife-69312-fig1-figsupp1-data2.pdf]

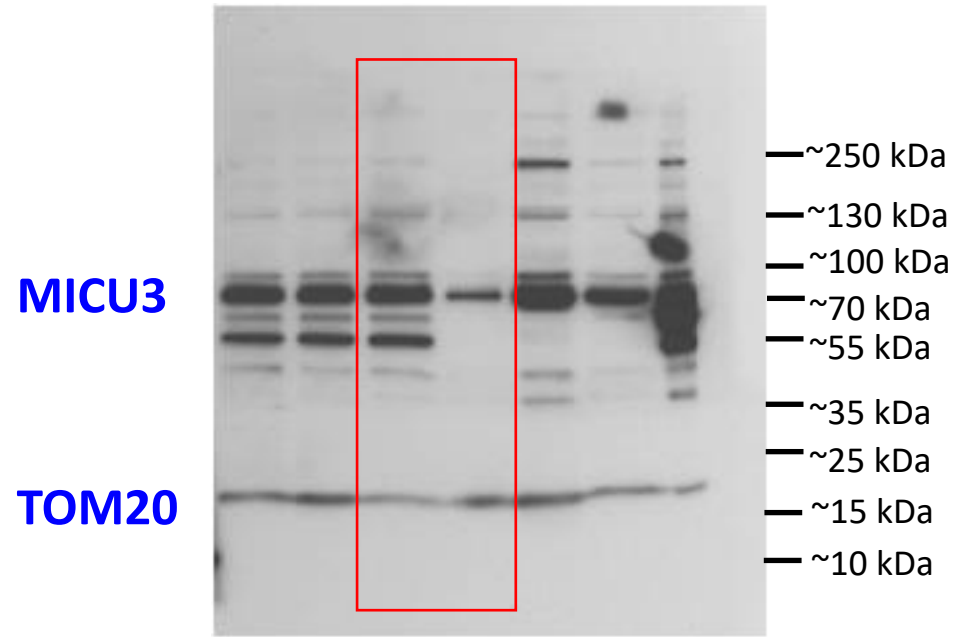

Figure 1—figure supplement 1—source data 3. Raw Western blot image for panel I.

Supplement: Figure 1—figure supplement 1—source data 3. [file elife-69312-fig1-figsupp1-data3.pdf]

MICU1

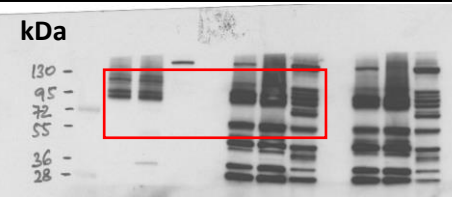

MCU

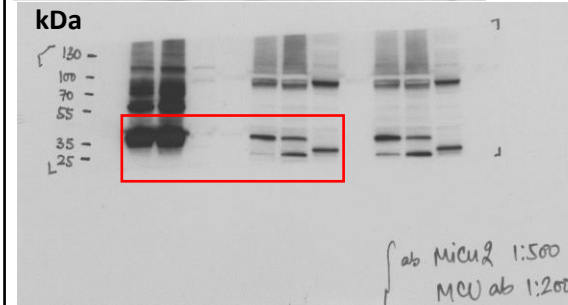

EMRE

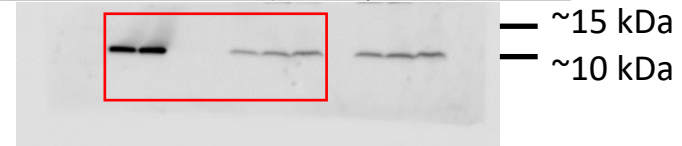

MICU2

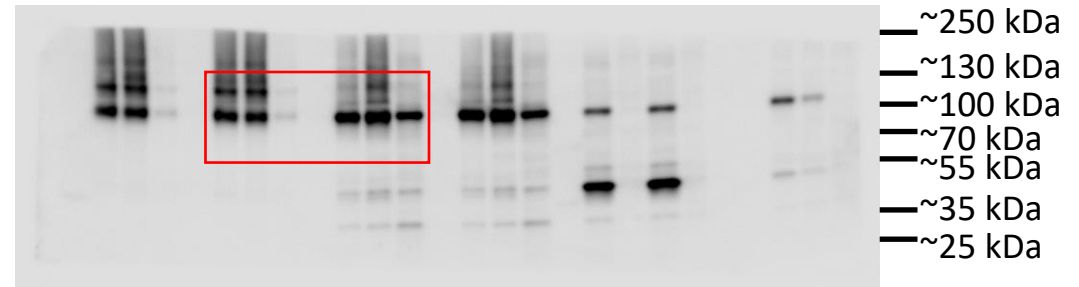

TOM20

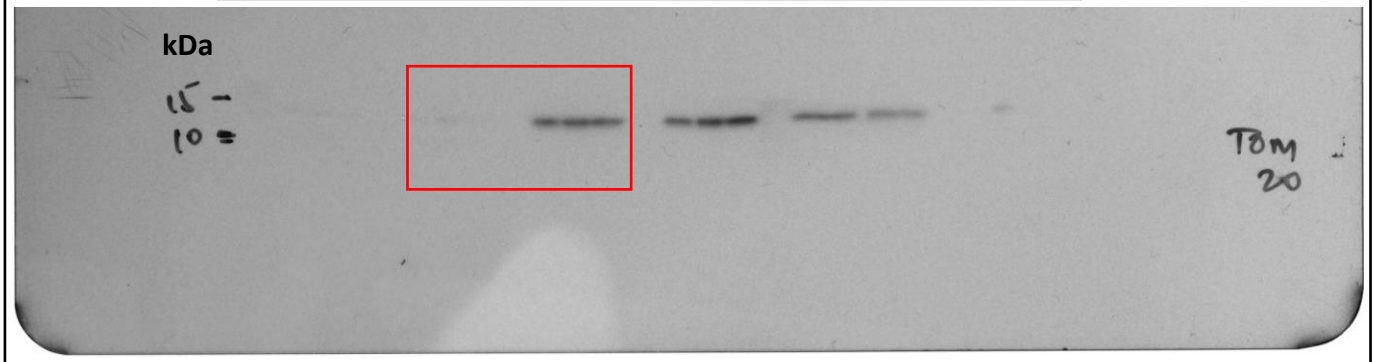

Figure 1—figure supplement 2—source data 4.  
Raw Western blot image for panel F.

Supplement: Figure 1—figure supplement 2—source data 1. [file elife-69312-fig1-figsupp2-data1.pdf]

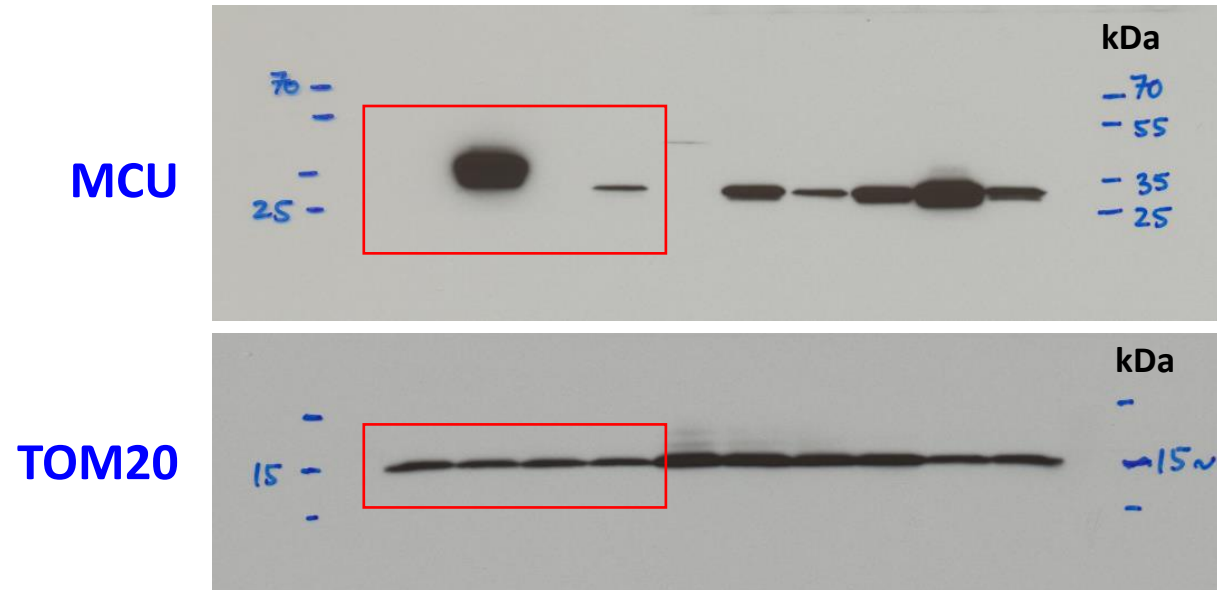

Figure 1—figure supplement 2—source data 5. Raw Western blot image for panel G.

Supplement: Figure 1—figure supplement 2—source data 2. [file elife-69312-fig1-figsupp2-data2.pdf]

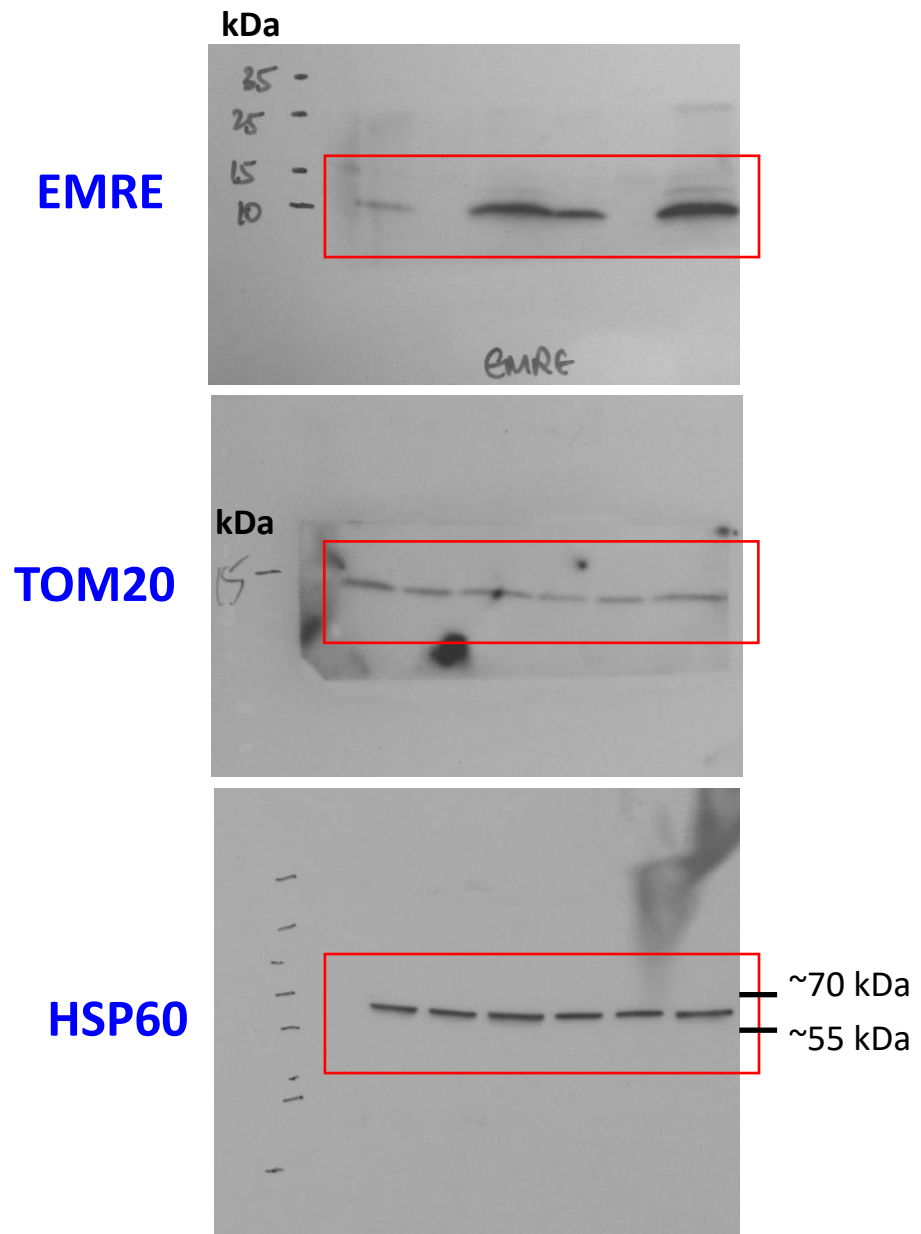

Figure 1—figure supplement 2—source data 6. Raw Western blot image for panel H.

Supplement: Figure 1—figure supplement 2—source data 3. [file elife-69312-fig1-figsupp2-data3.pdf]

**MICU1**

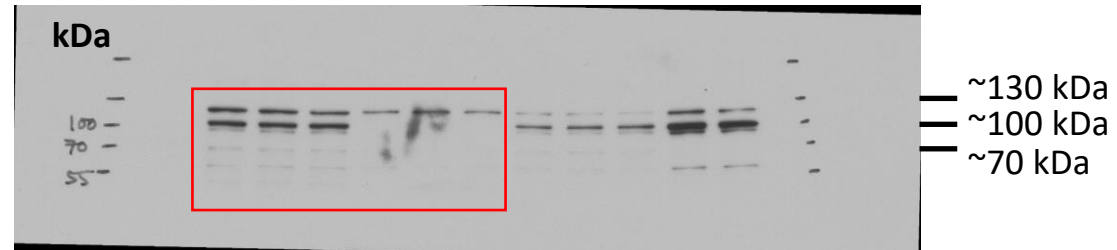

**EMRE**

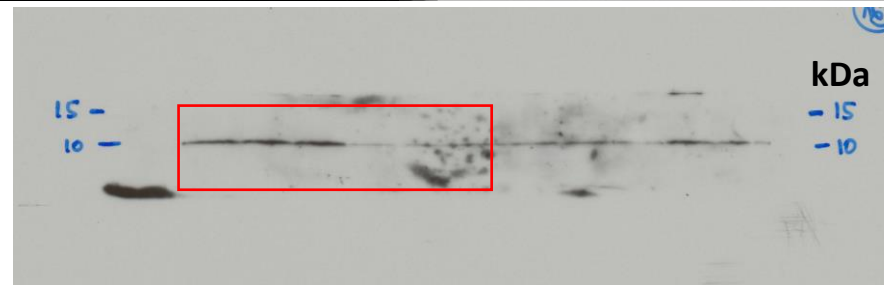

**HSP60**

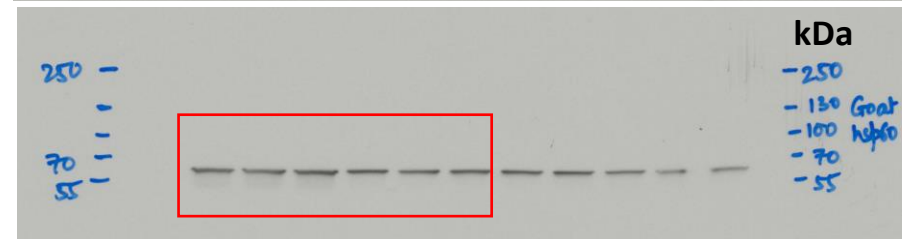

Figure 2—figure supplement 1—source data 1. Raw Western blot image for panel D.

Supplement: Figure 2—figure supplement 1—source data 1. [file elife-69312-fig2-figsupp1-data1.pdf]

HSP60

MCU

TOM20

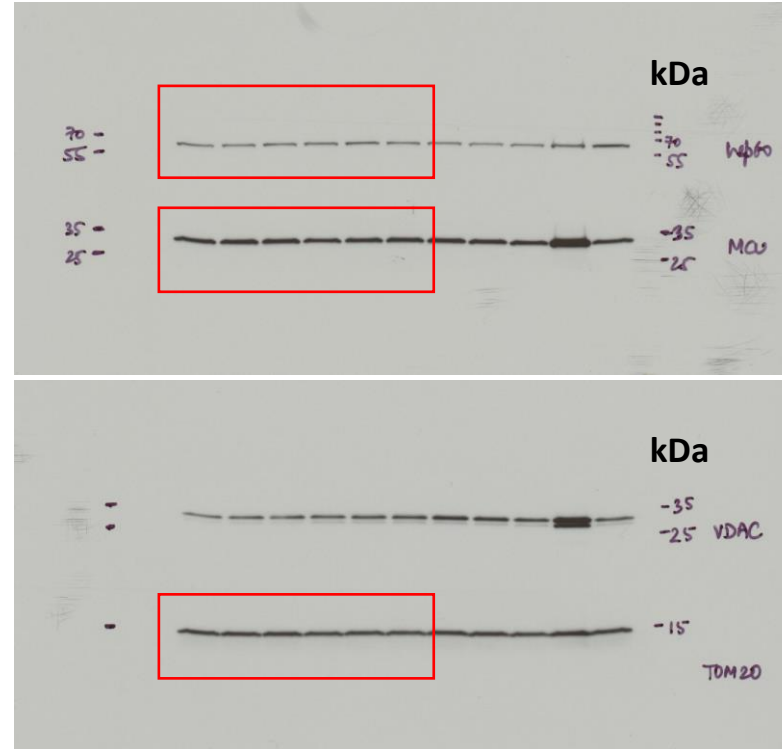

Figure 2—figure supplement 1—source data 2. Raw Western blot image for panel E.

Supplement: Figure 2—figure supplement 1—source data 2. [file elife-69312-fig2-figsupp1-data2.pdf]

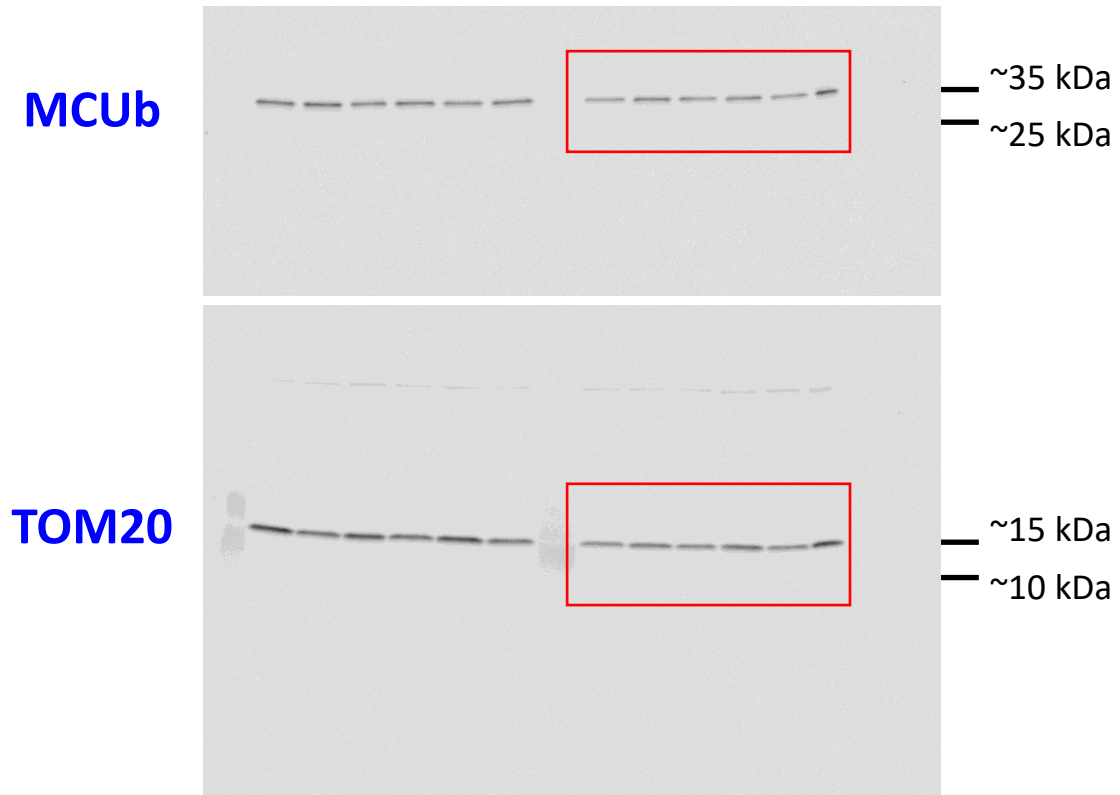

Figure 2—figure supplement 1—source data 3. Raw Western blot image for panel F.

Supplement: Figure 2—figure supplement 1—source data 3. [file elife-69312-fig2-figsupp1-data3.pdf]

**EMRE**

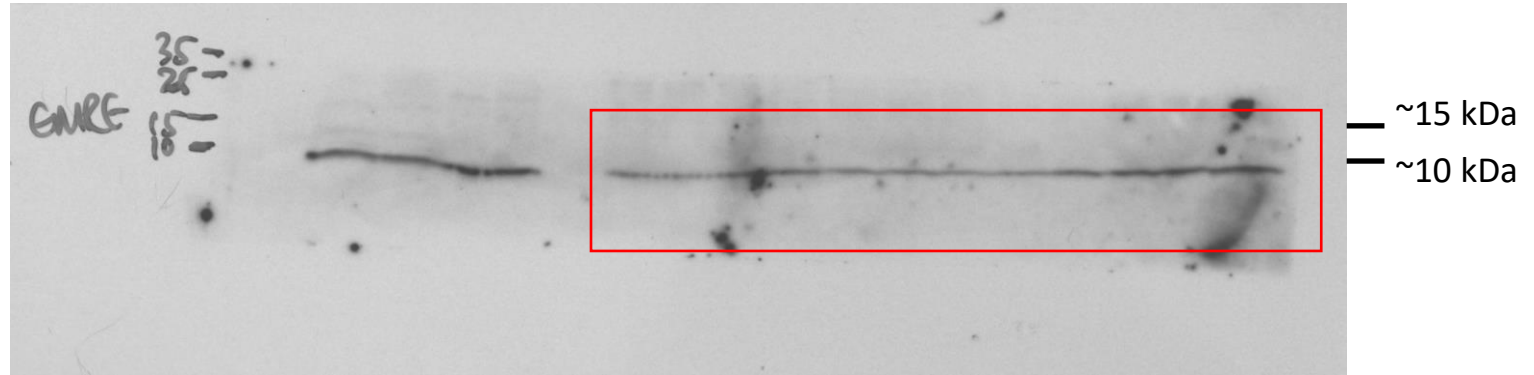

**TOM20**

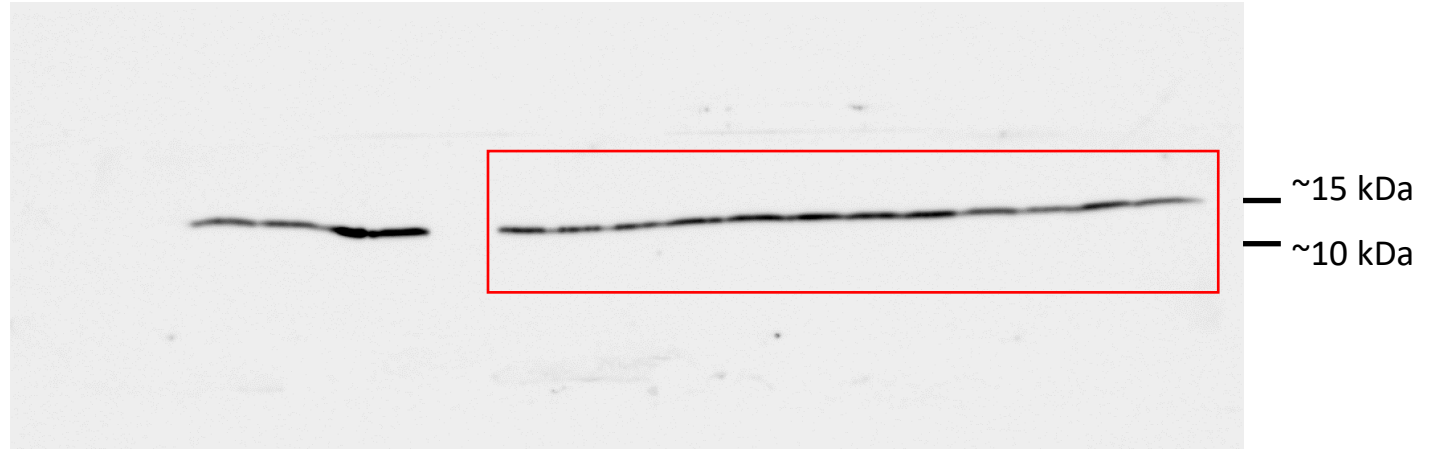

Figure 2—figure supplement 1—source data 4. Raw Western blot image for panel G.

Supplement: Figure 2—figure supplement 1—source data 4. [file elife-69312-fig2-figsupp1-data4.pdf]

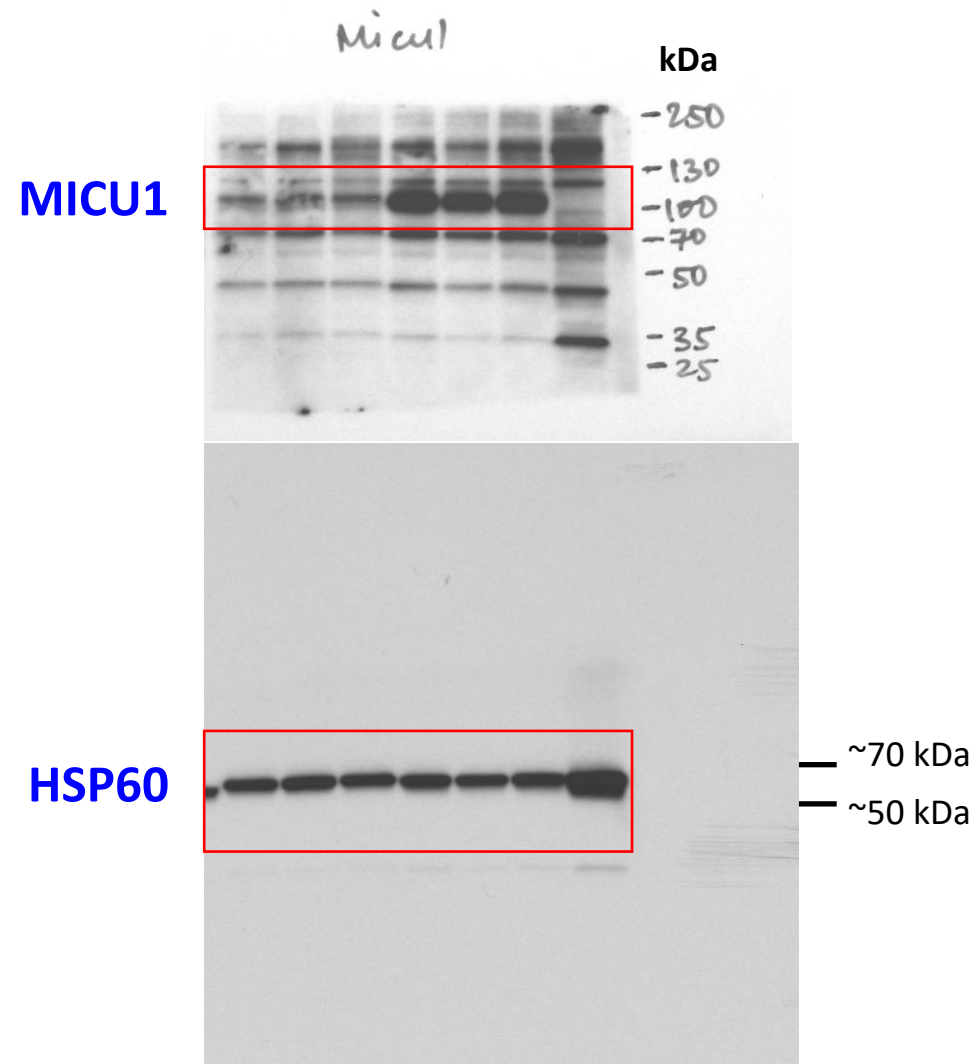

Figure 3—figure supplement 1—source data 1. Raw Western blot image for panel A (*Upper*).

Supplement: Figure 3—figure supplement 1—source data 1. [file elife-69312-fig3-figsupp1-data1.pdf]
