# Supplementary material for: The mechanism of MICU-dependent gating of the mitochondrial Ca2+uniporter
Source: eLife. 2021 Aug 31;10:e69312. doi: 10.7554/eLife.69312 (PMC8437439; doi:10.7554/eLife.69312)
Supplement: Figure 3—source data 1. [file elife-69312-fig3-data1.pdf]

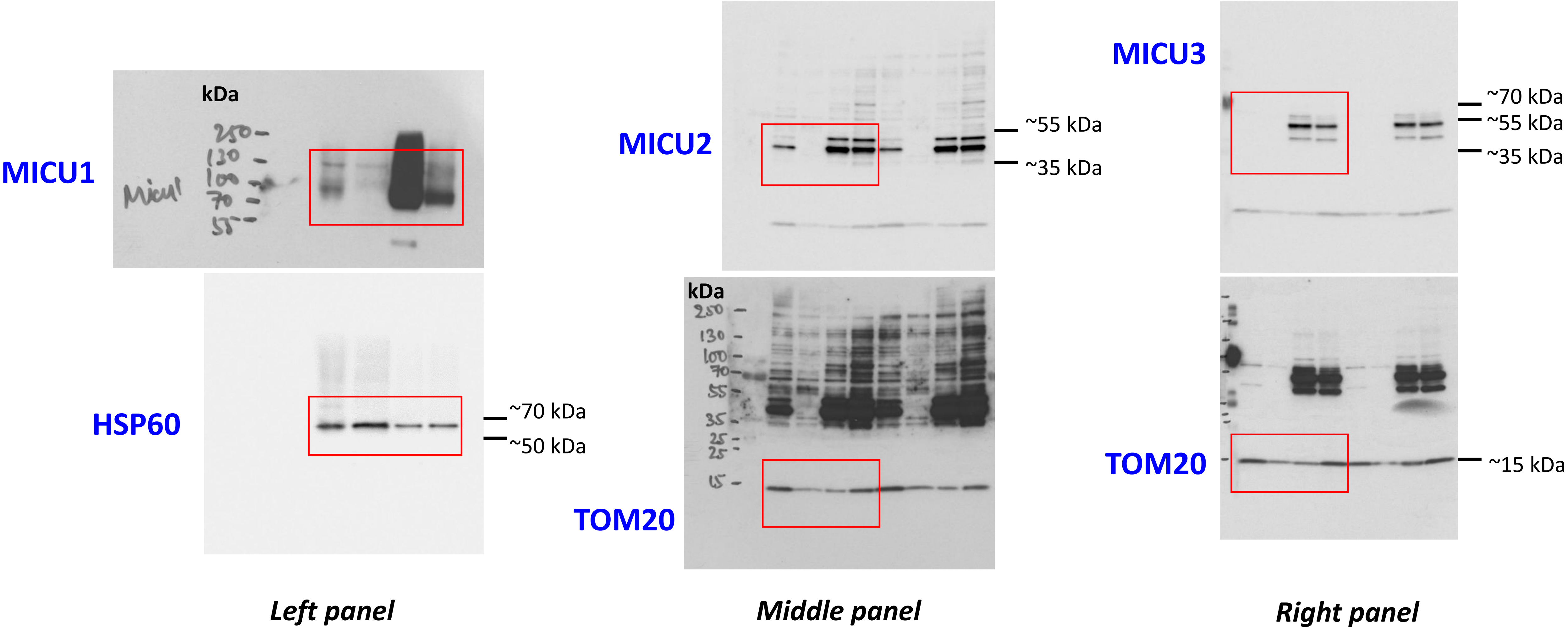

Figure 3—source data 1. Raw Western blot image for panel A.
