# Supplementary material for: The mechanism of MICU-dependent gating of the mitochondrial Ca2+uniporter
Source: eLife. 2021 Aug 31;10:e69312. doi: 10.7554/eLife.69312 (PMC8437439; doi:10.7554/eLife.69312)
Supplement: Figure 3—figure supplement 1—source data 2. [file elife-69312-fig3-figsupp1-data2.pdf]

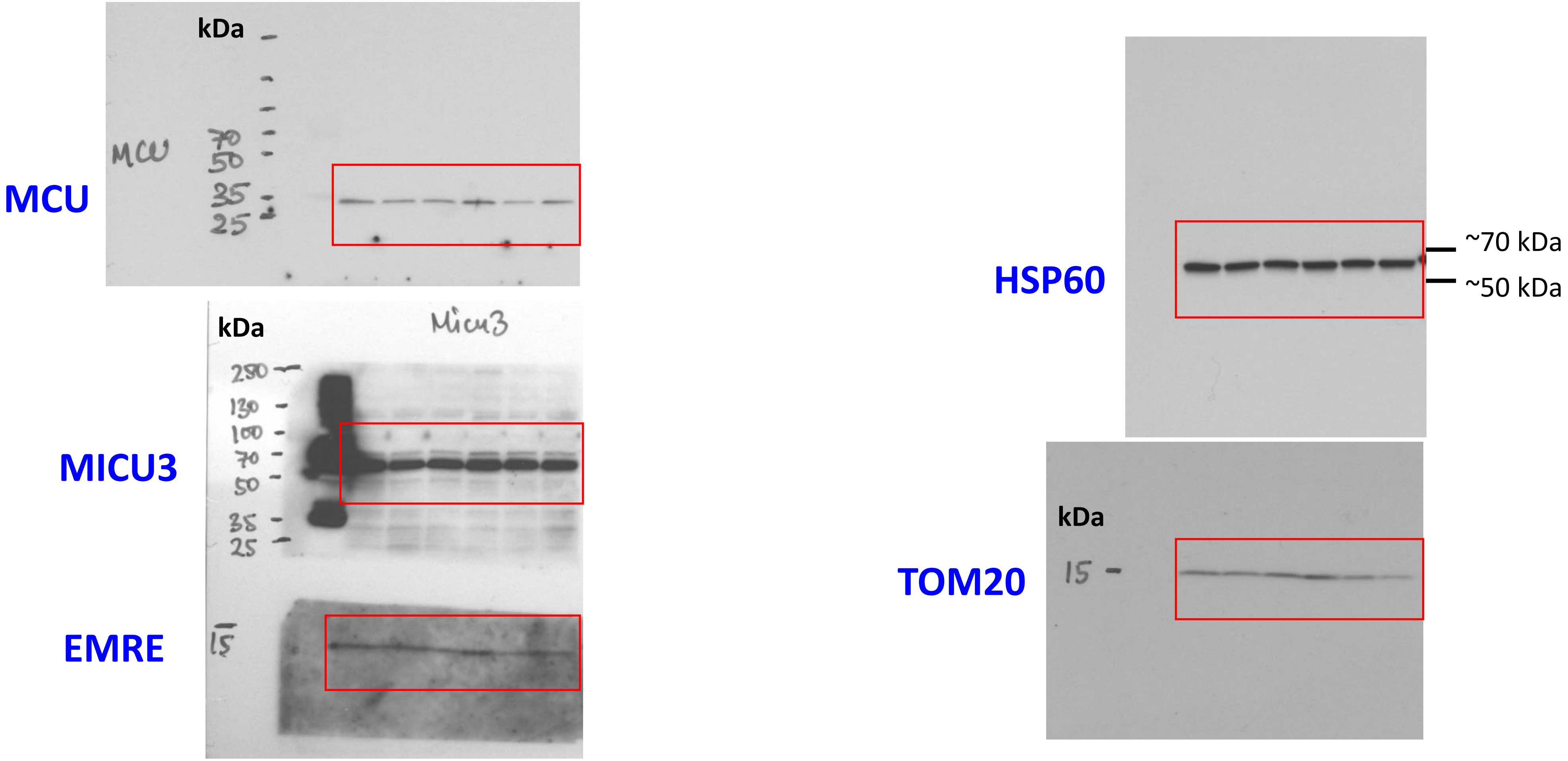

Figure 3–figure supplement 1—source data 2. Raw Western blot image for panel A (*Lower*).
