# Supplementary material for: The mechanism of MICU-dependent gating of the mitochondrial Ca2+uniporter
Source: eLife. 2021 Aug 31;10:e69312. doi: 10.7554/eLife.69312 (PMC8437439; doi:10.7554/eLife.69312)
Supplement: Supplementary file 1. — (a) MICU1 effect on MCUcx as determined by previous electrophysiological experiments. (b) MICU1 effect on MCUcx as determined by previous Ca2+ imaging experiments. [file elife-69312-supp1.docx]

**Supplementary File1a. MICU1 effect on MCU_cx_ as determined by previous electrophysiological experiments.**

| **Citation** | **MCU current** | |
| --- | --- | --- |
|  | **Low [Ca^2+^]_cyto_** | **High [Ca^2+^]_cyto_** |
| (Hoffman et al., 2013) |  | Inhibition |
| (Patron et al., 2014)  Lipid bilayer experiments;  EMRE subunit, essential for MCU activity and MICU1 interaction, was absent | No effect | Activation |
| (Vais et al., 2016) |  | No change |
| (Kamer et al., 2018) |  | No change |

**Supplementary File1b. MICU1 effect on MCU_cx_ as determined by previous Ca^2+^ imaging experiments.**

| **Reference** | **Effect of MICU1 on**  **mitochondrial Ca^2+^ uptake** | |
| --- | --- | --- |
|  | **Low [Ca^2+^]_cyto_** | **High [Ca^2+^]_cyto_** |
| (Mallilankaraman et al., 2012) | Inhibition | No effect |
| (Hoffman et al., 2013) | Inhibition | Inhibition |
| (Plovanich et al., 2013) |  | Activation |
| (Csordas et al., 2013) | Inhibition | Activation |
| (de la Fuente et al., 2014) | Inhibition | Activation |
| (Kamer and Mootha, 2014) | Inhibition |  |
| (Logan et al., 2014) | Inhibition | No effect |
| (Patron et al., 2014) | Inhibition | Inhibition |
| (Hall et al., 2014) |  | Inhibition |
| (Antony et al., 2016) | Inhibition | Activation |
| (Liu et al., 2016) | Inhibition | Activation |
| (Bhosale et al., 2017) | Inhibition |  |
| (Kamer et al., 2017) | Inhibition |  |
| (Paillard et al., 2018) | Inhibition | Activation |
| (Phillips et al., 2019) | Inhibition |  |

**REFERENCES**

Antony, A.N., Paillard, M., Moffat, C., Juskeviciute, E., Correnti, J., Bolon, B., Rubin, E., Csordas, G., Seifert, E.L., Hoek, J.B., et al. (2016). MICU1 regulation of mitochondrial Ca^2+^ uptake dictates survival and tissue regeneration. Nat Commun *7*, 10955.

Bhosale, G., Sharpe, J.A., Koh, A., Kouli, A., Szabadkai, G., and Duchen, M.R. (2017). Pathological consequences of MICU1 mutations on mitochondrial calcium signalling and bioenergetics. Biochim Biophys Acta Mol Cell Res *1864*, 1009-1017.

Csordas, G., Golenar, T., Seifert, E.L., Kamer, K.J., Sancak, Y., Perocchi, F., Moffat, C., Weaver, D., de la Fuente Perez, S., Bogorad, R., et al. (2013). MICU1 controls both the threshold and cooperative activation of the mitochondrial Ca^2+^ uniporter. Cell metabolism *17*, 976-987.

de la Fuente, S., Matesanz-Isabel, J., Fonteriz, R.I., Montero, M., and Alvarez, J. (2014). Dynamics of mitochondrial Ca^2+^ uptake in MICU1-knockdown cells. Biochem J *458*, 33-40.

Hall, D.D., Wu, Y., Domann, F.E., Spitz, D.R., and Anderson, M.E. (2014). Mitochondrial calcium uniporter activity is dispensable for MDA-MB-231 breast carcinoma cell survival. PLoS One *9*, e96866.

Hoffman, N.E., Chandramoorthy, H.C., Shamugapriya, S., Zhang, X., Rajan, S., Mallilankaraman, K., Gandhirajan, R.K., Vagnozzi, R.J., Ferrer, L.M., Sreekrishnanilayam, K., et al. (2013). MICU1 motifs define mitochondrial calcium uniporter binding and activity. Cell Rep *5*, 1576-1588.

Kamer, K.J., Grabarek, Z., and Mootha, V.K. (2017). High-affinity cooperative Ca^2+^ binding by MICU1-MICU2 serves as an on-off switch for the uniporter. EMBO Rep *18*, 1397-1411.

Kamer, K.J., and Mootha, V.K. (2014). MICU1 and MICU2 play nonredundant roles in the regulation of the mitochondrial calcium uniporter. EMBO Rep *15*, 299-307.

Kamer, K.J., Sancak, Y., Fomina, Y., Meisel, J.D., Chaudhuri, D., Grabarek, Z., and Mootha, V.K. (2018). MICU1 imparts the mitochondrial uniporter with the ability to discriminate between Ca^2+^ and Mn^2+^. Proc Natl Acad Sci U S A *115*, E7960-E7969.

Liu, J.C., Liu, J., Holmstrom, K.M., Menazza, S., Parks, R.J., Fergusson, M.M., Yu, Z.X., Springer, D.A., Halsey, C., Liu, C., et al. (2016). MICU1 Serves as a Molecular Gatekeeper to Prevent In Vivo Mitochondrial Calcium Overload. Cell Rep *16*, 1561-1573.

Logan, C.V., Szabadkai, G., Sharpe, J.A., Parry, D.A., Torelli, S., Childs, A.M., Kriek, M., Phadke, R., Johnson, C.A., Roberts, N.Y., et al. (2014). Loss-of-function mutations in MICU1 cause a brain and muscle disorder linked to primary alterations in mitochondrial calcium signaling. Nat Genet *46*, 188-193.

Mallilankaraman, K., Doonan, P., Cardenas, C., Chandramoorthy, H.C., Muller, M., Miller, R., Hoffman, N.E., Gandhirajan, R.K., Molgo, J., Birnbaum, M.J., et al. (2012). MICU1 is an essential gatekeeper for MCU-mediated mitochondrial Ca^2+^ uptake that regulates cell survival. Cell *151*, 630-644.

Paillard, M., Csordas, G., Huang, K.T., Varnai, P., Joseph, S.K., and Hajnoczky, G. (2018). MICU1 Interacts with the D-Ring of the MCU Pore to Control Its Ca^2+^ Flux and Sensitivity to Ru360. Mol Cell *72*, 778-785 e773.

Patron, M., Checchetto, V., Raffaello, A., Teardo, E., Vecellio Reane, D., Mantoan, M., Granatiero, V., Szabo, I., De Stefani, D., and Rizzuto, R. (2014). MICU1 and MICU2 finely tune the mitochondrial Ca^2+^ uniporter by exerting opposite effects on MCU activity. Mol Cell *53*, 726-737.

Phillips, C.B., Tsai, C.W., and Tsai, M.F. (2019). The conserved aspartate ring of MCU mediates MICU1 binding and regulation in the mitochondrial calcium uniporter complex. eLife *8*.

Plovanich, M., Bogorad, R.L., Sancak, Y., Kamer, K.J., Strittmatter, L., Li, A.A., Girgis, H.S., Kuchimanchi, S., De Groot, J., Speciner, L., et al. (2013). MICU2, a paralog of MICU1, resides within the mitochondrial uniporter complex to regulate calcium handling. PLoS One *8*, e55785.

Vais, H., Mallilankaraman, K., Mak, D.D., Hoff, H., Payne, R., Tanis, J.E., and Foskett, J.K. (2016). EMRE Is a Matrix Ca^2+^ Sensor that Governs Gatekeeping of the Mitochondrial Ca^2+^ Uniporter. Cell Rep *14*, 403-410.
